# Association of uncoordinated sucking pattern with developmental outcome in premature infants: a retrospective analysis

**DOI:** 10.1186/s12887-019-1811-1

**Published:** 2019-11-14

**Authors:** You Gyoung Yi, Byung-Mo Oh, Seung Han Shin, Jin Yong Shin, Ee-Kyung Kim, Hyung-Ik Shin

**Affiliations:** 1 Department of Rehabilitation Medicine, Seoul National University Hospital, Seoul National University College of Medicine, 101 Daehak-ro, Jongno-gu, Seoul, 03080 Republic of Korea; 2 Division of Neonatology, Department of Pediatrics, Seoul National University Children’s Hospital, 101 Daehak-ro, Jongno-gu, Seoul, 03080 Korea; 3 Division of Occupational Therapy, Department of Rehabilitation Medicine, Seoul National University Boramae Medical Center, Seoul, South Korea

**Keywords:** Premature infant, Feeding behavior, Neonatal Oral-motor assessment scale, Neurodevelopment

## Abstract

**Background:**

Stress signals during sucking activity such as nasal flaring, head turning, and extraneous movements of the body have been attributed to incoordination of sucking, swallowing, and respiration (SSR) in premature infants. However, the association of uncoordinated sucking pattern with developmental outcomes has not yet been investigated. The aim of this study was to investigate whether uncoordinated sucking pattern during bottle-feeding in premature infants is associated with the developmental outcomes at 8–12 and 18–24 months of age (corrected for prematurity).

**Methods:**

We retrospectively reviewed the medical records and video recordings for the Neonatal Oral-Motor Assessment Scale (NOMAS) of premature infants and divided them into two groups based on the presence or absence of incoordination. The Bayley-III cognition composite scores of the incoordination-positive and incoordination-negative group were compared at 8–12 and 18–24 months of age.

**Results:**

Seventy premature infants exhibited a disorganized sucking pattern according to the NOMAS. The average Bayley-III cognition composite scores at 8–12 months of age were 92.5 ± 15.6 and 103.0 ± 11.3 for the incoordination-positive (*n* = 22) and incoordination-negative groups (*n* = 48), respectively (*p* = 0.002). The average Bayley-III cognition composite scores at 18–24 months were 90.0 ± 17.9 and 100.7 ± 11.5 for the incoordination-positive (*n* = 21) and incoordination-negative groups (*n* = 46), respectively (*p* = 0.005). A multiple linear regression analysis indicated that the presence of uncoordinated sucking pattern, grade 3 or 4 germinal matrix hemorrhage–intraventricular hemorrhage, and moderate to severe bronchopulmonary dysplasia were independently associated with cognitive development at 18–24 months of age.

**Conclusions:**

Uncoordinated sucking pattern in premature infants was independently associated with a higher risk of abnormal developmental outcome in the cognitive domain of the Bayley-III at both 8–12 and 18–24 months. There may be a need for periodic follow-up and early intervention for developmental delay when incoordination of SSR that results in stress signals on the NOMAS is observed before 40 weeks postmenstrual age.

## Background

Feeding skills in a newborn requires complex functions that are directed by the central nervous system (CNS), including the coordination of sucking, swallowing, and respiration (SSR) [1–4]. Preterm infants often have difficulty in coordinating SSR [5–8]. It is reported that incoordination of SSR is associated with a longer transition time to full oral feeding in premature infants with tube feeding [9]. Additionally, lack of muscle strength and/or endurance could precipitate the inability to sustain sucking in premature infants [10].

Two hypotheses exist regarding the difficulty in sucking encountered by neonates: (i) CNS immaturity that is self-limiting as the child ages and (ii) neurologic dysfunction due to disruption of the CNS [1, 3, 11–14]. Distinguishing between these two potential causes of sucking difficulty is important to adequately counsel parents and commence early intervention to address developmental issues.

The Neonatal Oral-Motor Assessment Scale (NOMAS) is a noninvasive assessment tool to distinguish a dysfunctional or disorganized sucking pattern from a normal sucking in clinical practice [15, 16]. A dysfunctional sucking pattern in NOMAS is defined as abnormal jaw and tongue movements that result in the interruption of feeding, such as a flaccid tongue, excessive excursion of the jaw, or no movement. It is rarely observed [17] but is frequently accompanied by congenital anomalies, severe brain lesions, and neuromuscular disease [14, 18]. Palmer, the developer of the NOMAS system, suggested that a dysfunctional sucking pattern is strongly associated with neurological dysfunction, rather than CNS immaturity [18]. However, despite the high prevalence of a disorganized sucking pattern on NOMAS among premature infants [19], its association with developmental outcome is still unclear.

Previous studies have suggested that some items belonging to the disorganized sucking patterns could be more relevant to neurologic dysfunction [19, 20]. Recently, the Dutch NOMAS working group categorized disorganized sucking pattern in the original NOMAS into three clusters according to the presence of an arrhythmical sucking pattern, inability to sustain sucking, and incoordination of SSR, which results in stress signals [21]. However, each cluster does not consider the severity of sucking difficulty.

Palmer et al. [15] considered “incoordination of SSR that results in stress signals” in NOMAS to be reflected by nasal flaring, head turning, and extraneous movements of the body or limbs during sucking activity. The Dutch NOMAS working group added several symptoms, such as choking, coughing, gagging, yelping, and grunting, to the findings of stress signals. However, whether these symptoms are more relevant than other symptoms of sucking difficulty to developmental outcome is not yet known.

The aim of this study was to investigate whether uncoordinated sucking pattern during bottle feeding in premature infants is associated with the developmental outcomes at 8–12 and 18–24 months of age (corrected for prematurity).

## Materials and methods

### Subjects

We performed a retrospective review of the medical records of premature infants born between January 2014 and December 2016 at Seoul National University Hospital (SNUH) and referred for consultation to the Division of Pediatric Rehabilitation for sucking difficulty. The results of the NOMAS evaluation were incorporated into the electronic medical records, which were analyzed retrospectively by the authors. We included those infants who were born very premature (< 32 weeks) or very low birth weight (BW) (< 1500 g), assessed as having a disorganized sucking pattern in the NOMAS before 40 weeks postmenstrual age (PMA), and evaluated using the cognitive domain of the Bayley Scale of Infant Development, third edition (Bayley-III) [22], at 8–12 months of age (corrected for prematurity). Premature infants with the absence of Bayley-III at 8–12 months of age or the evaluated NOMAS after 40 weeks PMA were excluded. All procedures performed in studies involving human participants were in accordance with the ethical standards of the institutional and/or national research committee and with the 1964 Helsinki Declaration and its later amendments or comparable ethical standards. Ethical approval was obtained from the SNUH Institutional Review Board (no. 1711–128-901).

### NOMAS evaluation

All infants born earlier than 32 weeks gestational age (GA) or less than 1500 g with feeding difficulty had been referred to the Division of Pediatric Rehabilitation, and an occupational therapist evaluated their NOMAS score within 72 h from commencement of feeding in the hospital where this study was conducted. The evaluation was performed by analyzing the video recording of sucking activity [4]. Video recording included a close-up lateral view of the mouth, jaw, and neck. Only bottle-feeding was analyzed. The video recording started before the lip reached the bottle nipple and was stopped after recording for more than 2 min since the initiation of the oral feeding. Bottle-feeding was performed by a neonatal intensive care unit (NICU) nurse during the video recordings. Sucking difficulties were categorized into normal, disorganized, and dysfunctional sucking pattern according to the classification of the original NOMAS version [15].

### Conversion to NOMAS cluster system

For this study, the disorganized sucking pattern was divided into three clusters (cluster 2, 3, or 4) according to the cluster system presented by the Dutch group, as shown in Table 1 [21]. The existing NOMAS video recordings were re-evaluated and reclassified according to the cluster system by a rehabilitation doctor (one of the coauthors of this paper) who has been certified by Marjorie Meyer Palmer, the original developer of the NOMAS. The occupational therapist, who had more than 5 years of experience in rehabilitative interventions for swallowing function in infants at the NICU, also blindly evaluated the NOMAS cluster using the same video recordings. The first evaluator (rehabilitation physician) was blinded to clinical factors, but not to diagnosis, GA, and PMA. The second evaluator (occupational therapist) was blinded to all clinical factors.

**Table 1:** Scoring instructions and interpretation for each Neonatal Oral-Motor Assessment Scale cluster (reproduced with permission from Yi et al. [9])

Cluster	Interpretation	Scoring Instruction
1	Normal sucking pattern	
2	Disorganized sucking pattern	Only an arrhythmical sucking pattern, without the observation of “unable to sustain” or “incoordination of suck/swallow and respiration” sucking patterns
3	Disorganized sucking pattern	An arrhythmical and “unable to sustain” suckle pattern.
		The “unable to sustain” suckle pattern includes the following:
		1. The infant ceases sucking completely during the first 2 min of nutritive sucking, or
		2. The pauses are longer than the burst, or
		3. The bursts are shorter than three sucking phases
4	Disorganized sucking pattern	An arrhythmical and “incoordination of suck/swallow and respiration” sucking pattern that causes stress signals; the “unable to sustain” suckle pattern may or may not be present.
		“Incoordination of suck/swallow and respiration” includes all the following stress signals: nasal flaring, head turning, head bobbing, extraneous movements of the body or limbs, gagging, choking, coughing, yelping, and grunting.
5	Dysfunctional sucking pattern	The interruption of sucking activity owing to abnormal movements of the tongue and jaw which includes the following:
		1. Excessively wide excursions of the jaw, or
		2. Minimal excursions: clenching, or
		3. Flaccid tongue with absent tongue groove, or
		4. Retracted tongue with posterior humping

### Medical records acquisition

Premature infants included in this study were evaluated using the Bayley Scales of Infant Development, third edition (Bayley-III), cognition composite score at 8–12 and 18–24 months of age (corrected for prematurity). In addition to 18–24 months of corrected age, 8 to 12 months corrected age was chosen because it is known to be a good time to identify the suspicion or presence of cerebral palsy or other neurologic abnormality [23]. It is also known to be an appropriate time for the first developmental assessment to be performed, usually the Bayley Scales of Infant Development, because the children show little stranger anxiety at this age and most are cooperative [23].

Through a retrospective electrical medical record review, GA, sex, brain sonographic finding (such as germinal matrix hemorrhage–intraventricular hemorrhage [GMH-IVH], PMA at initiation of oral feeding (defined as oral consumption of at least 5 mL of milk), PMA at the time of NOMAS evaluation, small for gestational age (SGA) or appropriate for gestational age (AGA), severity of bronchopulmonary dysplasia (BPD), birth weight (BW), development of sepsis, and 5 min Apgar score were obtained.

### Statistical analysis

Cohen’s kappa was obtained for the interrater reliability of the two evaluators. The cognitive domain of Bayley-III was compared between the incoordination-positive (cluster 4) and incoordination-negative groups (clusters 2 and 3) at 8–12 and 18–24 months of age using the independent t-test. Pearson’s correlation coefficient r was calculated between continuous variables (GA and BW) and cognition composite score at 18–24 months. Multiple linear regression was performed with the Bayley-III cognition composite score at 18–24 months as the dependent variable. Analyses were performed using IBM SPSS version 20 software (IBM Corp., Armonk, NY, USA). A probability value < 0.05 was considered significant in all analyses.

## Results

### Subjects

A total of 71 premature infants had both a Bayley-III cognitive domain and NOMAS score. One premature infant belonging to cluster 5, who had a grade 3 GMH-IVH and was a cardiopulmonary resuscitation survivor, showed delayed cognitive development at 8–12 months of age and was excluded from the analysis since the infant did not belong to the disorganized sucking pattern in the NOMAS cluster system. Clinical characteristics of the premature infants included in the analysis (*n* = 70) are shown in Table 2. Among the premature infants with a disorganized sucking pattern in NOMAS, there were four premature infants in cluster 2, 44 premature infants in cluster 3, and 22 premature infants in cluster 4.

**Table 2:** Characteristics of premature infants (*n* = 70) included in the final analysis

Variable	Total (*n* = 70)	Incoordination-positive group (*n* = 22)	Incoordination-negative group (*n* = 48)	*p* Value
Sex				
Male	33 (47.14)	13 (59.09)	20 (41.67)	
Female	37 (52.86)	9 (40.91)	28 (58.33)	0.175
GA at birth (weeks)	29.0 ± 2.3	27.6 ± 2.1	29.6 ± 2.1	0.001
Birth weight (g)	1124.7 ± 344.5	952.3 ± 346.1	1203 ± 316.9	0.006
PMA at NOMAS evaluation (weeks)	34.8 ± 1.5	34.9 ± 1.7	34.8 ± 1.5	0.757
Apgar score (5 min)	6.6 ± 1.9	5.9 ± 1.8	6.9 ± 1.9	0.032
SGA	8 (11.4)	4 (18.2)	4 (8.3)	0.229
Moderate to severe BPD	23 (32.9)	12 (54.5)	11 (22.9)	0.009
GMH-IVH grades 3–4	4 (5.7)	1 (4.5)	3 (6.3)	0.775
Sepsis	8 (11.4)	7 (31.8)	1 (2.1)	<0.001

Values are presented as n (%) or mean ± standard deviation

*GA* gestational age, *PMA* postmenstrual age, *NOMAS* Neonatal Oral-Motor Assessment Scale, *SGA* small for gestational age, *BPD* bronchopulmonary dysplasia, *GMH-IVH* germinal matrix hemorrhage–intraventricular hemorrhage

### Interrater reliability for the NOMAS cluster system

The two evaluators agreed on the cluster levels for 65 of 70 recordings (Cohen’s kappa = 0.853). Disagreement occurred between clusters 2 and 3 (1 infants) and between clusters 3 and 4 (4 infants). The reliability of the two evaluators on the presence of incoordination items (cluster 4 vs. clusters 2 or 3) was higher with a Cohen’s kappa value of 0.864, with four disagreements being documented.

### Bayley-III cognition composite score at 8–12 and 18–24 months according to the incoordination findings in NOMAS

**Fig. 1:**
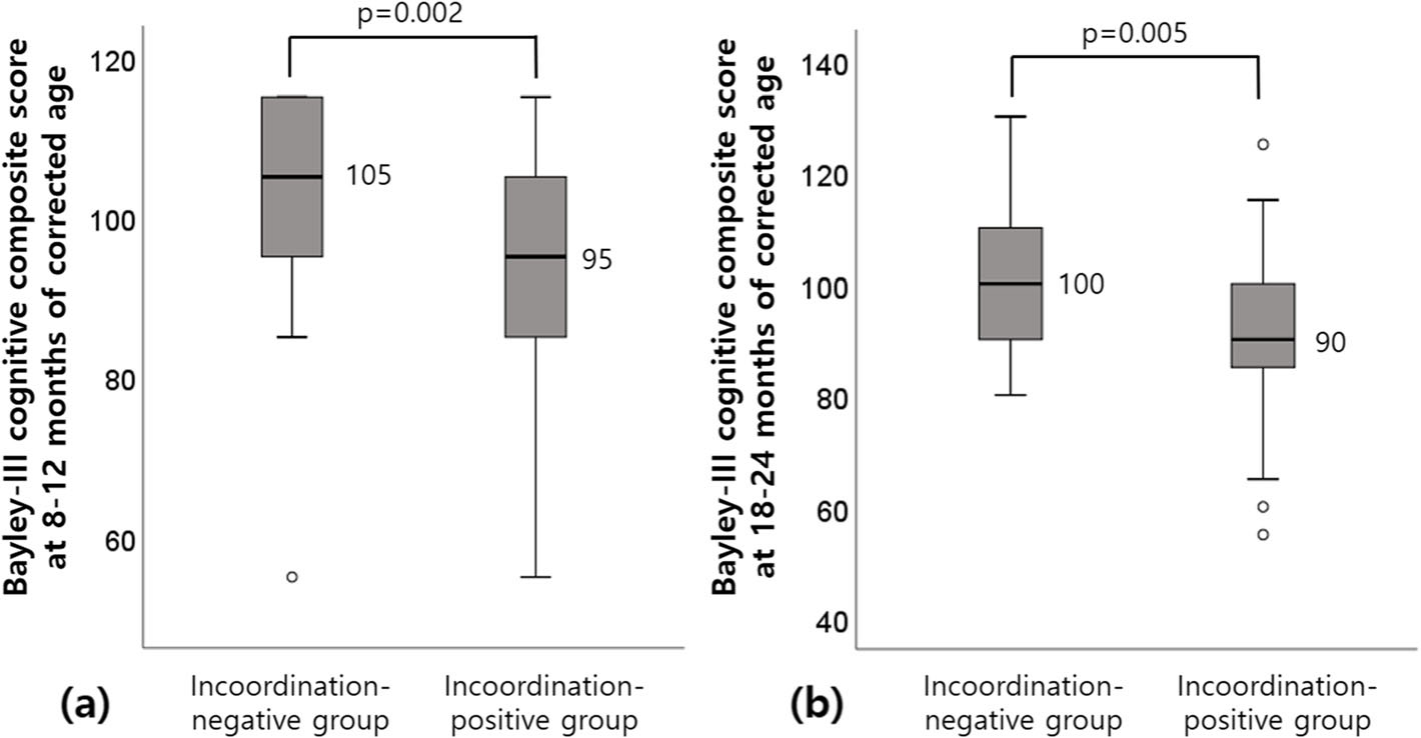
Bayley-III cognition composite score based on incoordination findings on the Neonatal Oral-Motor Assessment Scale. **a** At 8–12 months of age (corrected for prematurity). **b** At 18–24 months of age (corrected for prematurity). The median Bayley-III cognition composite score of each group is represented

The average Bayley-III cognition composite scores at 8–12 months were 92.5 ± 15.6 and 103.0 ± 11.3 for the incoordination-positive (*n* = 22) and incoordination-negative groups (*n* = 48), respectively (*p* = 0.002; Fig. 1a). The average Bayley-III cognition composite scores at 18–24 months were 90.0 ± 17.9 and 100.7 ± 11.5 for the incoordination-positive (*n* = 21) and incoordination-negative groups (*n* = 46), respectively (*p* = 0.005; Fig. 1b).

### Baseline characteristics related to cognitive development at 18–24 months

Independent t-test was performed to investigate the baseline characteristics related to the Bayley-III cognition composite score at 18–24 months (Table 3). The Bayley-III cognition composite scores at 18–24 months were different according to the presence of SGA, moderate to severe BPD, uncoordinated sucking pattern (incoordination-positive group vs. incoordination-negative group; cluster 4 vs. clusters 2 or 3), and grades 3 or 4 GMH-IVH. GA (R = 0.312, *p* = 0.010) and 5-min Apgar score (R = 0.333, *p* = 0.006, for Spearman’s correlation test) were found to be significantly correlated with Bayley-III cognition composite score at 18–24 months. All clinical variables were included in the multiple linear regression analysis model with stepwise selection to control multicollinearity between independent variables. In the multiple linear regression analysis, the R^2^ value of the final model was 0.331, and the variables included in this model were the presence of uncoordinated sucking pattern, grades 3 or 4 GMH-IVH, and moderate to severe BPD (Table 4).

**Table 3:** Bayley-III cognition composite score at 18–24 months of corrected age according to baseline characteristics of the premature infants (*n* = 67)

		n	Mean	SD	*p* Value
Respiratory distress syndrome	Yes	49	95.2	15.3	0.049
	No	18	103.1	10.7	
Small for gestational age	Yes	8	85.0	13.9	0.010
	No	59	99.0	13.9	
Moderate to severe BPD	Yes	23	89.1	16.5	0.001
	No	44	101.6	11.5	
Necrotizing enterocolitis	Yes	4	88.8	4.8	0.240
	No	62	97.6	14.8	
Sepsis	Yes	8	90.0	17.3	0.131
	No	59	98.3	14.0	
Pulmonary hypertension	Yes	6	95.8	21.8	0.796
	No	61	97.5	13.9	
Incoordination findings in NOMAS	Yes	21	90.0	17.9	0.005
	No	46	100.7	11.5	
Grades 3 or 4 GMH-IVH	Yes	3	71.7	14.4	0.001
	No	64	98.5	13.5	

*SD* standard deviation, *BPD* bronchopulmonary dysplasia, *NOMAS* Neonatal Oral-Motor Assessment Scale, *GMH-IVH* germinal matrix hemorrhage–intraventricular hemorrhage

**Table 4:** Multiple linear regression analysis: Bayley-III cognition composite score at 18–24 months of corrected age

Variable	B	95% CI for B	Beta	t	p Value
Incoordination-positive group on NOMAS	−7.737	−14.521 to −0.952	−0.249	−2.528	0.026
Grade 3 or 4 GMH-IVH	−23.818	− 38.371 to −9.265	−0.341	−3.271	0.002
Moderate to severe BPD	−8.477	−15.178 to − 1.775	−0.279	− 2.528	0.014

*CI* confidence interval, *NOMAS* Neonatal Oral-Motor Assessment Scale, *GMH-IVH* germinal matrix hemorrhage–intraventricular hemorrhage, *BPD* bronchopulmonary dysplasia

## Discussion

Premature infants frequently experience difficulty in sucking, thereby delaying the transition from tube to full oral feeding [24–26]. According to the NOMAS cluster system, sucking difficulty can be classified as arrhythmical sucking pattern, inability to sustain sucking pattern, incoordination of SSR sucking pattern, and dysfunctional sucking pattern (Table 1). Although the dysfunctional sucking pattern is known to be associated with poor developmental outcome, the relationship with developmental outcome is not well known in other patterns. The results of this study suggest that incoordination of SSR sucking pattern could be more associated with the development in the cognitive domain both at 8–12 and 18–24 months than arrhythmical sucking or inability to sustain pattern.

Most of the preterm infants with an immature sucking pattern can successfully bottle-feed as the SSR matures, and the sucking pattern of term infants is characterized by the rhythmic alternation of suction and expression/compression [11]. In the arrhythmical or inability to sustain pattern group, it could be assumed that SSR coordination has already been formed in preterm period, but sucking difficulty has occurred due to lack of oromotor strength and endurance [11], and this group showed a faster development than the group with less developed SSR coordination.

Previous studies have attempted to predict developmental delay through neonatal oromotor function. In a study of 27 premature infants without brain lesions, the risk of developmental delay increased when the premature infants exhibited a disorganized sucking pattern at 37 weeks PMA [20]. However, this study only examined the presence of disorganized sucking pattern and did not distinguish between specific patterns among disorganized sucking pattern.

The results of this study are compatible with those of the study by Nieuwenhuis et al. [19], who classified the disorganized sucking patterns in the NOMAS into two categories: disorganized due to arrhythmic sucking and disorganized due to lack of coordination of SSR. They concluded that uncoordinated sucking patterns, but not arrhythmic sucking patterns, were associated with abnormal fidgety movement at 14 weeks post-term [19]. However, in the previous study, they did not discern dysfunctional sucking pattern from incoordination sucking pattern in the NOMAS. Additionally, the study did not distinguish the inability to sustain sucking pattern separately.

Regarding brain ultrasound finding, GMH-IVH grades 3 and 4 are widely considered predictors of developmental delay, whereas the implications of GMH-IVH grades 1 and 2 remains controversial [27, 28]. In concert with previous studies, GMH-IVH grades 3 and 4 were identified as predictors of the cognition composite score in a multiple linear analysis in the present study. In the present study, BPD was also analyzed as a statistically significant predictor of developmental delay. Mizuno and colleagues reported that infants with BPD demonstrated not only poorer feeding coordination, but also poorer feeding endurance and performance [29], which might have affected development [30].

In the NOMAS, stress signals, including nasal flaring, head turning, and extraneous movement, are regarded as the symptoms of incoordination of SSR (Table 1), as described by Palmer et al. [16, 18]. However, the relationship between incoordination of SSR and stress signals was suggested through clinical observation and not by direct measurement of SSR. To demonstrate the incoordination of SSR, recordings of intraoral pressure (rhythmic alternation of suction and expression/compression) [11, 24], pharyngeal pressure [24, 25], nasal thermistor flow [26, 30], and thoracoabdominal plethysmography [24] have been used, but these methods are not widely used, particularly in the clinical settings, due to their complexity and invasiveness. However, in terms of research, the relationship between SSR incoordination and clinically observed stress symptoms could be investigated by directly measuring SSR. There might be certain stress signals that are more relevant to SSR incoordination than other signals.

There are a few limitations to this study. First, we did not include more objective signs that could be assessed in premature infants with sucking difficulty, including episodes of desaturation, apnea, and bradycardia. Those measurable signs might complement NOMAS, which is composed of observational findings. Second, because this study was conducted retrospectively, we only evaluated the sucking pattern before 40 weeks PMA since most premature infants are discharged from the NICU before term age. In our study, clusters 2 and 3 (i.e., arrhythmia and inability to sustain sucking without stress signals) were less relevant to neurodevelopmental outcomes. However, if those symptoms persist during the post-term period, the clinical relevance could be changed. For example, Wolthuis-Stigter et al. [10] reported that the inability to sustain sucking at 46 weeks significantly increased the odds of abnormal neurodevelopmental outcomes at 2 years of age. Therefore, for future studies, a prospective study design would be necessary for improving our understanding of the clinical significance of sucking difficulties that are observed in premature infants.

## Conclusion

Uncoordinated sucking pattern that results in stress signals (e.g., head bobbing, extraneous movements of the body or limbs during sucking, choking, gagging, coughing, yelping, and grunting) in premature infants was independently associated with a higher risk of abnormal developmental outcome in the cognitive domain both at 8–12 and 18–24 months. There might be a need for periodic follow-up and early intervention for developmental delay when incoordination of SSR that results in stress signals in NOMAS is observed before 40 weeks PMA.

## Data Availability

The datasets during and/or being analyzed during the current study are available from the corresponding author on reasonable request.

## Abbreviations

BPD: bronchopulmonary dysplasia
BW: birth weight
CA: corrected age
GA: gestational age
GMH-IVH: germinal matrix hemorrhage–intraventricular hemorrhage
NICU: neonatal intensive care unit
NOMAS: Neonatal Oral-Motor Assessment Scale
PMA: post-menopausal age
SGA: small for gestational age
SNUH: Seoul National University Hospital
SSR: sucking, swallowing, and respiration
